# Successful task shifting: a mixed-methods cross-sectional evaluation of an emergency obstetric care program to increase access to cesarean sections in rural Nepal

**DOI:** 10.1080/16549716.2024.2429888

**Published:** 2024-12-03

**Authors:** Rita Thapa Budhathoki, Abigail G. Knoble, Suresh Tamang, Bal Sundar Chansi Shrestha, Arpana BC Kalaunee, Indra Rai, Bikash Shrestha, Pravin Paudel, Ruma Rajbhandari, Archana Amatya

**Affiliations:** a Research Advocacy, and Monitoring, Nick Simons Institute, Lalitpur, Nepal; b Mass General Gastroenterology, Mass General Hospital, Boston, MA, USA; c Mass General Hospital, Division of Global Health Equity, Boston, MA, USA

**Keywords:** Health systems, emergency surgery, human resources, cesarean section, global surgery, Nepal, South Asia, rural health

## Abstract

**Background:**

Direct obstetric causes of maternal mortality account for approximately 86% of all global maternal deaths. In Nepal, 12% of all deaths of women of reproductive age are due to preventable obstetric complications in significant part due to the limited distribution and skill level of human resources.

**Objectives:**

To address this, the Advanced Skilled Birth Attendant (ASBA) task-shifting initiative was developed to train medical officers to perform Cesarean sections (CSs) and manage obstetric emergencies in Nepal. Until now, there has been limited study of the program’s efficacy.

**Methods:**

A survey targeting all 234 ASBA graduates resulted in 93 usable surveys for multivariate regression. Additionally, seven rural government hospitals with ASBA graduates were selected for 13 in-depth interviews and 6 focus group discussions. Results were then triangulated.

**Results:**

Immediately after training, 92.7% of ASBA graduates reported performing CSs, with the majority (65.6%) continuing to perform CSs today. Of the ASBAs not performing CSs, 51.7% could be explained by the lack of a functional operating theater, despite being at hospitals expected to provide CSs. ASBAs were significantly more likely to be performing CSs with a family physician or another ASBA present (*p* < 0.001; *p* < 0.001). Their work was perceived to increase the use of services, improve access, reduce out-referrals, and reduce the burden of CSs on any one staff member.

**Conclusions:**

The ASBA program successfully reduces human resource shortages, expands the provision of life-saving Cesarean section, and improves the working conditions in rural hospitals within the LMIC setting.

## Paper context

**Main findings:** When stationed at lower-level health facilities with functional operating theaters, Medical Officers trained to perform Cesarean sections effectively utilized their skills, addressing the human resource shortage contributing to maternal and child mortality in low-resource settings, improving access to life-saving surgical care, reducing the burden of Cesarean sections on individual staff members, facilitating a positive working environment, and decreasing the need for out-referrals from lower-level hospitals.

**Added knowledge:** This research expands the evidence for task-shifting programs in regions beyond sub-Saharan Africa and provides information on the Cesarean sections done by Non-physician Clinicians and the wider impacts on the working environment where they are stationed.

**Global health impact for policy and action:** These results provide support for national task-shifting initiatives to both increase access to life-saving surgical care, but also to improving the wider working environment at health facilities, which may contribute to positive health care worker retention and overall improved access to Cesarean sections.

## Background

In 2020, approximately 800 women died every day from preventable causes related to pregnancy and child-birth, the majority in low- and middle-income countries (LMICs) [1]. Despite significant strides in improving maternal health outcomes, direct obstetric causes of maternal mortality still account for approximately 86% of all global maternal deaths, the majority of which are preventable [2]. Nepal’s maternal mortality ratio (MMR) is still one of the highest in Asia and maternal deaths account for approximately 12% of all deaths among women of reproductive age due to preventable obstetric complications and a lack of specialized doctors [3,4]. As of the 2021 census, the MMR was 151 deaths per 100,000 live births, more than double the Sustainable Development Goal, and even higher in rural areas where 79% of the 30 million Nepalis reside [3,5].

Addressing life-threatening obstetric complications through improved clinical management, such as the provision of cesarean sections (CSs), predicts a 63% decrease in MMR in low-income countries by merely increasing CSs levels to those recommended by the World Health Organization (WHO) (10–15%) [6,7]. In 2016, rural Nepal had a crude CS rate of 5.9%, with some regions as low as 2.2%, significantly below the WHO’s recommendations, and likely inaccurate given that the rural institutional birth rate was estimated to be as low as 30% at the time [8,9]. Although urban rates of CSs have been increasing, particularly with the expansion of private hospitals, access to CSs remains a significant hurdle for women in rural Nepal [8,10].

The distribution of human resources (HR) and availability of healthcare workers capable of providing CSs in LMICs is a significant limiting factor to reducing MMR globally, and a significant problem in Nepal [11–13]. This has led the WHO to recommend ‘task shifting’ to increase access to life-saving medical procedures by optimizing the roles of less specialized healthcare workers to address the shortage and poor distribution of healthcare professionals [11]. Task shifting has been used globally to ensure access to essential healthcare despite the limited number of specialized HR for anesthesiology services, mental healthcare professionals, and maternal and newborn care [12,14].

In 2013, the Nick Simons Institute (NSI) partnered with the National Health Training Center (NHTC) of Nepal to develop the Advanced Skilled Birth Attendant (ASBA) task shifting program to train non-specialist medical officers (MOs) to provide safe access to CSs where they might not otherwise be available [15]. This was done as part of NSI’s wider physician-led generalist surgical team initiative to ensure access to surgery in rural parts of Nepal. These teams are composed of the MDGP (Medical Doctorate in General Practice), ASBA trained MOs, Anesthesia Assistants or non-doctor anesthesia providers, and OT-trained nurses who together address HR shortages preventing access to surgery in remote and geographically difficult places [13,16]. Although the ASBA program in the generalist surgical team model is unique to Nepal, similar cadres of MOs trained to perform CSs in similar contexts include Obstetric Medical Officers, non-physician clinicians (NPCs), or emergency surgery officers [17,18].

MOs have a bachelor’s degree covering anatomy, pharmacology, pathology, community health, medicine, and minor surgery and usually work under the supervision of a senior health professional, such as an MDGP, equivalent to a family physician [13,19]. The objective of the 70-day ASBA training is to enhance MOs’ clinical skills with regard to the management of obstetric emergencies, primarily focusing on the clinical skills needed for CS along with other core skills including shock in pregnancy, Robson criteria, ectopic pregnancy, and blood transfusions [20]. Following this, they are involved in assisting and/or performing basic and advanced cases under supervision until skill competency is achieved and the individual feels confident performing the procedure independently, carrying out a minimum of 15 CSs during their training [20]. As of 2023, there were four ASBA training sites, three of which are supported by NSI who aim to train 40 medical officers, while others are supported by the province. Participants do not pay for the training. ASBAs supported by NSI are bonded for one year after their graduation and are generally placed in rural hospitals in need of their skills. Although there is no strict government policy, some hospitals occasionally require trainees to commit to a certain period of service at their hospital on an ad hoc basis. Typically, ASBAs are placed in health facilities that offer Comprehensive Emergency Obstetric and Newborn Care (CEmONC) services with a functioning OT, along with a senior staff member, an Anesthesiologist, or more likely, an Anesthesia Assistant, as well as OT trained nurses. Although all government hospitals included in the study, from Primary to Tertiary, are directed to provide CEmONC, shortages in HR, nonfunctional equipment, and poor management result in a very different ground reality [21]. As of 2020 (Nepali Calendar Year: 2076/2077), around 234 ASBAs had been trained, after which graduates are supposed to have been deployed to CEmONC hospitals so that they can utilize their newly acquired skills [22]. As of 2023, there is no follow-up training.

Until now, there has been no formal study of the ASBA training program’s efficacy beyond training feedback. Thus, NSI conducted a mixed method, cross-sectional study to explore the working status of ASBA graduates. This study focuses on both hindering and facilitating factors contributing to ASBAs ability to successfully perform CSs and the extent to which the program has filled the gap in HR by providing comprehensive emergency obstructive care in rural Nepali hospitals. The overall aim of the study was to investigate the effectiveness of the ASBA task shifting program on the performance of CS and to identify both the hindering and facilitating factors that influence performance of CSs by ASBA in Nepal.

## Methods

### Study design

This study employed convergent parallel mixed methods allowing for the collection and analysis of both quantitative and qualitative data followed by triangulation to compare and contrast findings. This approach was selected to leverage the strengths of both methodologies, offering a comprehensive understanding of the ASBA program’s efficacy. The quantitative data provided statistical insights into ASBA demographics. working conditions, and facilitating and hindering factors nationally, while the qualitative data offered deeper contextual understanding of the perceptions of key stakeholders regarding the program’s impact. This allowed for a nuanced understanding of the quantitative results and provided insight to the *why* behind the *what*. Sampling was conducted independently for each part of the study. Hindering and facilitating factors were triangulated for both quantitative and qualitative data. Convergence and divergence of these themes were then analyzed.

### Setting

This study was conducted across multiple health facilities in Nepal. Quantitative data collection was performed online via an emailed questionnaire, while qualitative data collection involved on-site interviews and focus group discussions at selected rural hospitals. These hospitals were chosen to represent diverse geographic locations across all seven provinces in Nepal.

### Quantitative data study

A cross-sectional study design was used for the quantitative portion of the research. The study population included all 234 ASBAs who had graduated from the ASBA training program by the time of the survey. A structured questionnaire was emailed to the study population (Survey Monkey) and data was collected from 1 March 2022, to 31 May 2022. Variables included basic demographic information, information about both the health facility during ASBAs’ initial placement after training and the health facility at the time of the survey, information about the health facilities, their working conditions, other qualified staff at the health facility, who was primarily responsible for CSs, the number of CSs performed per month, and hindering and facilitating factors related to successfully performing CSs. ASBAs successfully performing CSs at their current health facility at the time of the survey was the primary outcome. When survey data appeared to be inconsistent, individuals were contacted for clarification. If they could not be reached the data was excluded from the analysis. *See Figure S1.*

Data was recorded in Excel and analyzed with StataSE 17. Fisher’s exact test was used for between-group comparisons of categorical variables, and univariate and multivariate logistic regressions were employed to identify independent factors associated with the primary outcome.

### Qualitative data study

**Figure 1.**
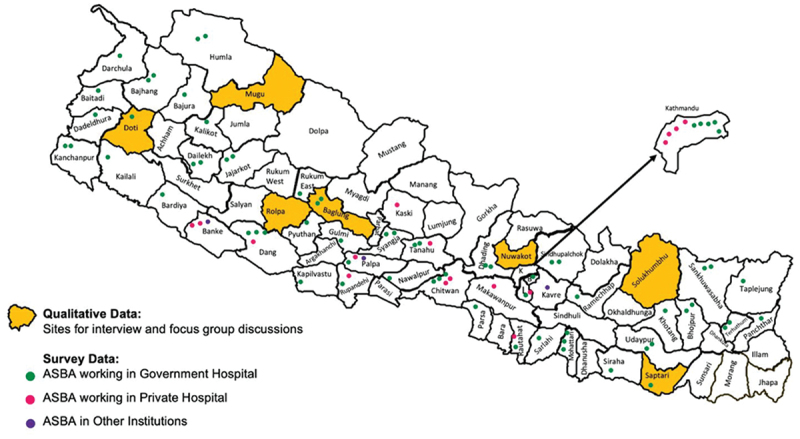
Distribution of ASBA survey respondents (*n*=93) and qualitative sites (*n*=7) by district.

An exploratory qualitative study design was implemented for the qualitative component of this study. The study participants included ASBAs, Medical Superintendents (Me.Su.s), and operating theater (OT) teams from selected hospitals. One hospital was chosen from each of the seven provinces independently from the survey respondents that either currently had an ASBA or had an ASBA in the past. There may have been overlap between the quantitative and qualitative participants but given that the survey was anonymous, the percentage of overlap was not quantifiable. All ASBAs at selected hospitals were included in the study. OT teams, and Me.Su.s were then interviewed based on availability. Semi-structured interviews with eight ASBAs, four Me.Su.s, and six focus group discussions with the OT team were conducted over 1–3 days during 1^st^ of February − 30^th^ of April 2022. Topic guides were developed in an iterative process throughout. The conversations were tape-recorded, transcribed, and translated into English. A sample was then back translated from English to Nepali as a quality check. See Figure 1 and Table 1.

**Table 1.** Characteristics of the qualitative interview sites (n = 7).

Health Facility Level	n	ASBA Present?	Offering CSs?	IDI with ASBAs	IDI with Me.Su.s	FGD with OT Team	TOTAL
Primary Health Center**	1	1/1	1/1	1	1	1	**3**
Primary	2	2/2	2/2	3	1	2	**6**
Secondary A	3	2/3	3*/3	3	1	3	**7**
Tertiary	1	1/1	1*/1	1	1	0	**2**
**TOTALS**	**7**	**6/7**	**7/7**	**8**	**4**	**6**	**18**

One health facility was selected from each of the seven provinces. IDI = in-depth discussion interview. FGD = Focus group discussion. *At these health facilities, CSs were being provided, but not by the ASBA. **The Primary Health Center was in the process of upgrading to a Primary Hospital and thus was able to offer CS service.

A hybrid thematic analysis approach was employed to analyze the qualitative data. A deductive framework was first applied to examine predefined hindering and facilitating themes for ASBAs performing CSs, while an inductive approach was used to identify additional themes that emerged directly from the data. The research team independently generated these new themes and reached consensus through discussion. The data was then coded according to the agreed themes in NVivo 12.

### Public involvement

While the general public was not involved in the design and conduct of this research, concerned stakeholders in the Ministry of Health of Nepal were consulted, particularly those at the NHTC involved with the ASBA training program, prior to undertaking this evaluation. Various stakeholders, involved with maternal and child health in Nepal, will be informed of the results through a policy brief that will be circulated for a non-specialist audience upon publication.

### Ethical approval and consent to participate

This study was approved by the Nepal Health Research Council (Reg. 18/2022P). *See Figure S2*. Participants under the age of 18 were not included. The survey respondents indicated their consent via a survey question. For the qualitative portion, study hospitals and interviewees received an information sheet and an oral explanation about the study after which they gave informed written consent. The authors did not receive grants directly supporting this work. The funders had no role in the study design, data collection and analysis, decision to publish, or preparation of the manuscript.

## Results

### Survey findings

Of the 234 graduates of ASBA training who were contacted via email, 47.9% (n = 112) responded to the survey, and 39.7% (n = 93) were usable for analysis. Respondent demographics are described in Table 2.

**Table 2.** Demographics of ASBA survey respondents (n = 93).

Category		n	(%)
Sex	Male	81	88.04
	Female	11	11.96
Age (years)	≤30	46	50.00
	31 - 40	44	47.83
	41 - 50	1	1.09
	≥50	1	1.09
Caste/ethnicity	Brahmin/Chhetri	43	46.74
	Janajati/Indigenous	21	22.83
	Dalit	5	5.43
	Other	23	25.00
Current degree	MBBS	77	83.70
	MDGP/family physician	12	13.04
	Other	3	3.26
Current Health Facility	Government: Primary	26	28.57
	Government: Secondary A	22	24.18
	Government: Secondary B	13	14.29
	Government: Tertiary	9	9.89
	Private/mission	17	18.68
	Other	4	4.40
Contract Type	Permanent	15	16.30
	Contract	77	83.70
Time in position (month)	mean ± s.d.	20.94 ± 17.43	
Are surgical services provided by your current health facility?	Yes	75	83.33
	No	15	16.67
Are cesarean sections provided by your current health facility?	Yes	70	89.74
	No	8	10.26
What staff is mostly involved in performing CSs at your current health facility?	ASBA	36	38.71
	MDGP/family physician	9	9.68
	OB/GYN	21	22.58
	All	5	5.38
	N/A	22	22.92
How often do you perform CSs?	Frequently	46	49.46
	Occasionally	13	13.98
	Rarely	2	2.15
	Never	11	11.83
	N/A	21	22.58
MDGP/family physician present at health facility	Yes	45	48.39
	No	48	51.61
OB/GYN present at health facility	Yes	30	32.26
	No	63	67.74
Another ASBA present at health facility	Yes	31	33.33
	No	62	66.67

Some responses were left blank from the demographic data and could not be reached for clarification, resulting in a slight variation of totals. Current health facility refers to the time at which the respondents completed the survey in 2022.

#### Initial health facility and working conditions

Following graduation, 92.7% of ASBA graduates reported performing CSs, completing a mean of 8.30 ± 6.35 CSs, ranging from 1 to 30 CSs, per month. The majority of graduates (85.5%) were initially placed at a Primary or Secondary A health facility on contract. A 71.8% reported performing CSs without direct supervision and 56.8% and 37.8% reported performing CSs ‘frequently’ and ‘occasionally’, respectively. *See Table S1 for Demographics of ASBAs at Initial Health Facilities.*

At some point following their ASBA graduation, 13.0% obtained a higher medical degree. These individuals were included in the analysis to best examine the current practices of all ASBAs and the greater impact on task-shifting initiatives in the Nepali healthcare system. At their current positions, 75.0% are stationed as MOs, 9.8% as family physicians, 4.4% as surgeons, 2.2% as Me.Su.s, and 8.7% as ‘other’. Roughly half (51.6%) of survey respondents had changed health facilities since their first posting after the ASBA training.

#### Current health facility and working conditions

As of 2022, 65.6% of ASBA graduates are performing CSs, performing an average of 9.85 ± 8.14 CSs per month ranging from 1 to 37, showing an increase since ASBAs first placement after the training. A 88.9% of ASBAs who had not changed health facilities since their first posting were significantly more likely to be practicing CSs compared to only 43.8% those who changed health facilities (*p* < 0.001). Of those who perform CSs, 75.0% reported performing CSs without direct supervision and 73.3% described performing CSs ‘frequently’, 21.7% ‘occasionally’, and 3.3% ‘rarely’.

Of the 60 ASBAs performing CSs, 47 (78.3%) were placed at a health facility with a supervisor such as a family physician or OB/GYN. Thirteen (21.7%) were stationed without a senior staff member. Ten (16.7%) had another staff member present who could complete CSs, such as another ASBA, and 3 (5%) were the only staff at their health facility that could perform and were able to offer CSs. *See Table S1 and Table S2.*

**Figure 2.**
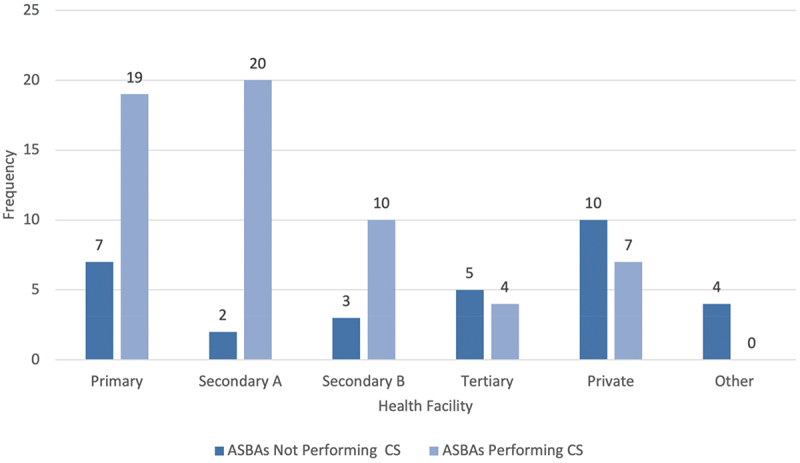
Health facilities of ASBA graduates performing and not performing CSs (*n*=91). 51.7% of ASBAs not performing CSs did not have a functional OT at their health facility, accounting for 8 out of 9 ASBAs not performing CSs at primary and secondary a health facilities.

Out of the 70 ASBAs working at any government hospital (Primary, Secondary A, Secondary B, or Tertiary), 75.7% were performing CSs at the time of the survey compared to only 33.3% of ASBAs at private/mission hospitals or other places of work. ASBAs signed as contract employees (83.7%) were significantly more likely to be performing CSs compared to those signed as permanent employees (p = 0.037). Initially, only ASBAs at Secondary A hospitals appeared to be performing CSs significantly more than at Tertiary hospitals (p = 0.012) (See Table 3). However, excluding the 16.9% of health facilities that did not have a functional OT, ASBAs at Primary and Secondary A health facilities were significantly more likely to be performing CSs when compared to Tertiary health facilities (*p* = 0.005; *p* = 0.001). The absence of a functional OT explained why 51.7% of all ASBAs were not performing CSs at their current health facility. This was especially true at Primary and Secondary A health facilities, where the lack of a functional OT explained 8 out of 9 ASBAs not utilizing their CS training, despite both hospital levels being expected to province CEmONC (See Figure 2). (21)

**Table 3.** Current working environments of ASBAs performing and not performing CSs (n = 92).

Category		Not Performing CS (*n*=32)	Performing CS (*n*=60)	*p* value
Sex	Male	27	54	0.506
	Female	5	6	
Age (years)	≤30	12	34	0.125
	31≤	20	26	
Caste/ethnicity	Janajati/Adibashi	10	11	0.588
	Dalit	2	3	1.000
	Other	3	14	0.219
	Brahmin Chhetri	16	27	ref
Time at facility (mo.)	Mean ± s.d.	16.22 ± 14.65	23.07 ± 18.27	0.090 *
Are surgical services provided by your current health facility?	Yes	14	61	<0.001 ***
	No	15	0	
Health Facility Level	Government: Primary	7	19	0.220
	Government: Secondary A	2	20	0.012 **
	Government: Secondary B	3	10	0.187
	Private/mission	10	7	1.000
	Government: Tertiary	5	4	ref
Contract Type	Permanent	9	6	0.037 **
	Contract	23	54	
MDGP/family physician present at health facility		4	41	<0.001 ***
OB/GYN present at health facility		10	20	0.806
Another ASBA present at health facility		2	29	<0.001 ***

At their current working environment. Some responses were left blank from the demographic data and could not be clarified, resulting in a slight variation of totals. Two-sided Fisher’s Exact Test run for *p* value. Two-sided t-test was run for the time at facility, df=70. *p*<0.10 *, *p*<0.05 **, *p*<0.001***.

A multivariate logistic regression was run with statistically significant predictive variables from the univariate analysis (Table 3). The greatest predictive variable as to whether an ASBA was performing CSs was the presence of a family physician (*p* < 0.001). Additionally, ASBAs with temporary contracts (*p* = 0.018), the presence of another ASBA at the health facility (*p* = 0.014), and longer time at the facility (*p* = 0.003), were significant predictors of ASBAs successfully performing CSs (Table 4).

**Table 4.** Multivariate analysis of ASBAs performing CS and Not performing CSs (n = 85).

Category		OR	95% C.I.	*p* value
Time at Facility (mo.)	Mean ± s.d.	1.080	1.023; 1.137	0.003***
Contract Type	Contract	27.651	1.776; 430.591	0.018*
Health Facility	Government: Primary	2.496	0.100; 62.056	0.577
	Government: Secondary A	3.694	0.087; 156.760	0.494
	Government: Secondary B	3.123	0.092; 106.043	0.527
	Private/mission	1.131	0.031; 40.926	0.947
	Government: Tertiary	ref		
MDGP/family physician present at health facility performing CSs		31.922	4.589; 222.052	<0.001***
ASBAs present at health facility performing CSs		17.686	1.791; 174.664	0.014*

At their current working location. Operating room presence was excluded as it was a perfect predictor of success. *p*<0.10 *, *p*<0.05 **, *p*<0.01 ***.

ASBAs were also asked about who they perceived to be primarily responsible for performing CSs at their current health facility and were more likely to perceive themselves as the staff primarily responsible for CSs if another ASBA was present (*p* = 0.010). Inversely, the presence of an OB/GYN decreased the likelihood that an ASBA would perceive themselves to be the staff primarily responsible for CSs as would be expected given the OB/GYN expertise (*p* = 0.032). *See supporting Table S3 and Table S4 for univariate and multivariate analysis of this secondary outcome.*

### Qualitative findings

Four overarching themes were identified in the qualitative data, and representative quotes were selected. The themes were: i) Beneficial Impacts of ASBA, ii) Facilitating Factors, iii) Hindering Factors, and iv) Recommended Improvements for the ASBA Training Program.

#### Beneficial impacts of ASBA

##### Increased access to care

ASBAs, Me.Su., and their OT teams perceived the program positively, describing reduced referrals, increased service access, and improved timeliness of referrals for more complicated cases in rural regions at the lower level hospitals.

I think this is a good approach in a country like Nepal because in remote places, it is almost impossible to get the service by Gynecologist … … .we have ASBA graduates and they are serving here, it is a pride for us. - Me.Su.

Last year, we had 5 neonatal deaths but now [since ASBA graduates working] there is not a single death. No mother has died due to an obstructive problem. Similarly, there are no referral cases this year. - MO/ASBA

##### Reduced referrals

One of the most frequently cited impacts of the ASBA program was reduced referrals to higher level hospitals and improved timeliness of referrals for more complicated cases at the lower level health facilities.

If the CS service is not available here then we have to refer to Jumla and it takes 7–8 hours to reach there due to bad roads and any bad thing can happen on the way. - OT Team

It is effective–there is no doubt. For example, after completion of training, I have not referred even a single case. It’s been 9 months. - MO/ASBA

##### Improved working environment

ASBAs themselves were described as competent and knowledgeable by the majority of their colleagues and supervisors. Having an ASBA at the hospital was seen to increase trust, facilitate a positive working environment, and improve maternal health outcomes. The working environment improved due to fewer HR limitations and a shared CS caseload. Hospital staff used words like ‘*happy*’, ‘*joyful*’, and ‘*pride*’ when describing how they felt offering expanded services.

If there were no ASBAs, I would have to look after the patient every day which could be very difficult (with my other responsibilities). There is ASBA so they are first on-call which makes it easy. - MDGP (family physician) doctor - OT Team

People’s trust in us has increased. Due to the availability of CS services, delivery flow and other services have been increasing. Overall there is a positive impact. - OT Team

#### Facilitating factors

Of the eight ASBAs interviewed, six were actively applying their ASBA training. Facilitating factors for the utilization of ASBA training included difficult and rural geography, a larger catchment area to maintain an adequate caseload, a lack of specialists, and having more than one ASBA or a senior staff member at a location to share the caseload and consult on complicated cases.

##### Rural areas with sufficient catchment area

Rural hospitals such as Doti and Mugu saw the benefit of the ASBA program and recommended it in other rural and geographically difficult areas with a sufficient catchment area.

Rural district hospitals where case flow is high should hire an ASBA. - OT Team

… Doti, Bajhang, and Bajura, every district situated in hills and mountains needs it [ASBA]. Even the terai districts where it takes time to reach district hospitals *…* - Me.Su.

##### Presence of a senior staff member or another ASBA graduate

The hospitals where ASBAs utilized their skills the most had another ASBA or a senior colleague such as a gynecologist or a family physician, which improved their confidence and suggested the presence of a functional OT. ASBAs described an increase in confidence with a supervisor or fellow ASBA graduate as they could rotate shifts and consult on difficult cases. In contrast, if there were too many senior staff, the ASBAs did not get the opportunity to utilize their skills at all.

There should be at least one [ASBA] backup with ASBA training. Otherwise, at least MDGP [family physician] should be there for six months on rotation. It is quite difficult to operate independently based on 70 days of ASBA training. - MO/ASBA,

#### Hindering factors

##### Level of hospital and the presence of a specialist team

Of the eight ASBAs interviewed, the two ASBAs not utilizing their training skills cited the presence of specialist teams (such as multiple gynecologists) as the primary reason they were not utilizing their training and regretted being unable to utilize their training. One of the Me.Su.s from a tertiary level hospital did not see any use for ASBAs, describing it as a ‘waste of resources’ in the tertiary hospital setting. This view was the exception to the otherwise positive perceptions of the ASBA program and was location specific in the higher-level hospitals. The COVID-19 pandemic also complicated their training process as hospitals shifted priorities towards the medical needs in the ER and COVID-19 wards.

ASBA training should not be provided to central tertiary hospitals which already have a few gynecologists. - Me.Su., Tertiary Hospital

I could not utilize my skills… as gynecology consultants are available in this hospital*…*. - MO/ASBA

##### Lack of infrastructure or equipment

Other factors that hindered the full use of ASBAs’ skills included poor case exposure in their placement site, lack of blood or a blood bank, and limited equipment. These factors echoed the general challenges faced by all the hospitals where interviews took place.

I had taken ASBA training while I was in Mugu, but the environment was not ready to conduct CS. There was no infrastructure for OT. So, we could not continue CS service after doing 4–5 cases. - ASBA on OT Team

Another challenge is a blood bank. When patients come from far away… when we cannot arrange blood, we feel sad *…* . - MO/ASBA

### Recommended improvements for ASBA training

#### ASBA training recommendations

Seven of the eight ASBAs had a positive experience with the training, describing it as ‘effective’ and ‘essential’. Limitations were tied to lower case exposure at their training hospital, either due to the hospital level, the presence of a specialist team, or the COVID-19 pandemic.

##### Better recruitment criteria

Multiple interviewees mentioned a minimum time commitment to ensure continuity of care and a return on investment. Although this was not directly mentioned at other hospitals, staff leaving after a short time was a challenge.

I think we should select doctors who can give at least two years after training. - Me.Su.

If a pre-assessment is taken then only an interested, dedicated person will apply. - OT Team

##### Curriculum suggestions

Curriculum suggestions included comprehensive abortion care (CAC), USG, vasectomy, hysterectomy, and repeat CSs due to scarring. Safe abortion care was the only curriculum-based suggestion that was repeated in the interviews (*n* = 3). Additionally, emergency hysterectomy was the most frequently requested (*n* = 12) to both expand services and increase confidence.

I would suggest adding the skills of abortion care. - MO/ASBA

One ASBA graduate mentioned having taken a USG training simultaneously with the ASBA training. She recommended this for other ASBA trainees as it made her more confident in her work and allowed her to diagnose issues earlier and promptly refer cases beyond her skill level.

If we have some USG component for some duration in ASBA training we can manage very easily. I did the USG assessment training simultaneously so that I am more confident. - MO/ASBA

##### Follow-up and refresher programs

Finally, follow-up and refresher courses were requested to keep ASBAs up to date with protocols, especially where they have a smaller caseload. This may help with confidence issues that ASBAs mentioned and long-term evaluation of ASBA skills retention and utilization.

It would be good to follow up with the ASBAs similar to mentoring programs for SBAs. Some skills may be forgotten. - OT Team

### Integrating quantitative and qualitative components

The quantitative portion of the study provided statistical insights about the general state of ASBAs across Nepal, shown in Table 5. There was convergence for facilitating factors including additional staff, such as the presence of a family physician or another ASBA at a lower level hospital. The qualitative data added depth to this, suggesting that a single OB/GYN had a similar facilitating effect of a family physician or another ASBA and it was the team of OB/GYNs that hindered ASBAs performing CSs. In contrast, the quantitative results alone showed the presence of an OB/GYN, with no reference to the number, was a hindering factor.

**Table 5.** Convergence and divergence quantitative and qualitative findings.

Factor	Quantitative Results	Qualitative Results	Summary
Facilitating Factors	*Additional staff*: Presence of a family physician Another ASBA	*Additional staff*: Presence of a family physician Another ASBA A single OB/GYN	*Convergence*: Both highlight the importance of a functional OT, additional staff, and lower level hospitals. *Divergence*: Qualitative results emphasized the importance in rural and remote regions while still having a large enough caseload to maintain skill level. Quantitative results showed that time at the facility and contract employees were predictors of ASBAs practicing CSs.
	Lower level hospitals (with a functional OT)	Lower level hospitals (with a functional OT)	
	Contract Employees	Rural and geographically remote	
	Longer time at the facility	Large enough caseload	
Hindering Factors	Higher level Hospitals	Higher level Hospitals	*Convergence*: Higher level hospital with specialist teams and the lack of a functional OT were found to be hindering factors. *Divergence*: While quantitative results were inconclusive about the presence of an OB/GYN, qualitative results explained that a single OB/GYN was facilitative – like an MDGP – multiple OB/GYNs were prohibitive.
	Lack of a functioning OT	Lack of a functioning OT	
		Specialist teams	

## Discussion

The Lancet Commission on Global Surgery estimates 5 billion people do not have access to safe and affordable surgical care, the majority of whom reside in LMICs where 9 out of 10 people do not have access to basic surgical care [23]. Despite holding 37% of the global population, a tragically disproportionate 6% of surgical procedures occur in the poorest countries [23]. A continuing challenge to providing access to surgery, including CSs, is the lack of trained healthcare professionals [24]. Thus, task shifting has been utilized to close this gap globally, and has become well established as a successful policy that alleviates medical HR shortages in LMICs [11,12,14,25–27]. The Advanced Skilled Birth Attendant (ASBA) program is a task-shifting initiative to increase maternal and newborn survival by training medical officers to perform CS and manage obstetric emergencies in Nepal. This paper’s parallel mixed methods approach supports the existing literature, showing that ASBA graduates are effective at providing CSs where they are otherwise unavailable, when placed in the right environments.

Following graduation, 92.7% of ASBA graduates reported performing CSs and the majority of ASBAs (65.6%) continue to provide CSs up to 10 years after their training, echoing the existing global literature where retention among NPCs was found to be higher than those of traditionally trained physicians [28]. A national Nepali newspaper reporting on a rural district hospital said, ‘*In the absence of specialist doctors’ … ‘medical officers have been conducting caesarean sections to save the lives of pregnant women. Specialist doctors refuse to be posted in Mugu due to its geographical remoteness*.’ [29]. Although ASBAs were not significantly more likely to be performing CSs at lower-level hospitals (Primary or Secondary A) as initially as predicted, when taking into consideration the presence of a functional operating theater, ASBAs at lower level hospitals were significantly more likely to be performing CSs (*p* = 0.005; *p* = 0.001). This is because the lack of a functioning operating theater accounted for 51.7% of all ASBAs not performing CSs and 8 out of 9 ASBAs not performing CSs at lower level hospitals despite the lower level government hospitals being required to provide CEmONC [21]. Similarly, a study on task shifting at first referral units in India found that non-‘operative facilities’ was the second most common barrier preventing emergency obstetric care services from being provided [30]. This further underscores the need for a holistic approach to clinical service provision in low resource settings and the need to place ASBAs at facilities that are actually able to provide CEmONC.

With more staff to provide access to CSs, staff reported that referrals out of lower level hospitals decreased, and more people were coming to their hospital for services after the placement of an ASBA graduate. This is supported in the literature, which found a significant increase in institutional birth rates from 30 to 77% when emergency obstetric care was implemented at both the hospital and lower-level facilities in rural Nepal [9,31]. In the same study, CSs remained only 3.8% of the births, despite a rapid rise in institutional birthrates [31]. Another rural Nepali hospital documented that three-quarters of performed CSs were emergency procedures, confirming that the increase in CS access is meeting the need for emergency CSs [32]. Although CS numbers were not directly recorded in this study, a similar task-shifting program in rural Zambia saw an increase in the number of CSs (+15.2%) in the intervention hospitals where NPCs were placed, suggesting that when available, the service is utilized [33].

Increased access to CSs in the hospitals was perceived by hospital staff to reduce the financial burden on the patient and increase the morale of the hospital. This echoed the findings of a similar task-shifting policy in Senegal where the work was perceived to be valuable and reduce the burden on patients [34]. This supports the efficacy of the ASBA training as a method of task shifting, improving access to lifesaving CSs, improving maternal and child health outcomes, and bolstering morale at health facilities where ASBAs were able to utilize their skills. A major theme in the qualitative interviews highlighted this, saying that the presence of an ASBA allowed for staff to share emergency CSs responsibilities, reduce burnout, improve the working environment of the hospital, and reducing the burden of CSs on any one staff member. Although patient outcomes were not directly measured in this study, other research has shown that there is no significant difference in maternal or fetal deaths between births attended by trained NPCs compared to traditionally trained physicians in multiple LMIC settings [28,35,36]. One major study in Tigray, Ethiopia retrospectively analyzed 25,629 deliveries and 11,059 obstetric procedures at 13 health facilities. NPCs carried out 63.3% of procedures, including 55.5% of the 2,835 CSs. There were no significant statistical differences in maternal deaths, fetal deaths, or length of hospital stay based on the type of attending staff. Additionally, physicians tended to perform elective CSs, whereas NPCs more frequently handled those necessitated by emergencies [36].

Contract ASBAs were also significantly more likely to be performing CSs compared to their permanent counterparts (*p* = 0.018). This supports previous findings, where contract or temporary Skilled Birth Attendant nurses were found to be more productive than permanent nurses in terms of the number of deliveries [37].

Ultimately, the ASBA program reduces the critical HR shortage in rural Nepal, improves access to life-saving medical treatment where it might not otherwise be available, reduces out-referrals, and distributes the responsibility of CSs between staff members, allowing for a more sustainable workload for healthcare providers long term. However, their skills could only be utilized when placed in functional CEmONC health facilities, and were more likely to be practicing in Primary and Secondary A hospitals where the HR shortage was more severe.

There would have been many maternal and neonatal deaths if there were no ASBAs. I have been working here at [redacted] District, which is one of the most remote districts of Nepal and I am proud of [the work I have done]. - ASBA respondent

## Strengths and limitations

This was a convergent parallel mixed-methods cross-sectional descriptive study, so no causation should be inferred. However, the use of mixed methods allowed for convergent data analysis, relying both on statistical analysis and nuanced experiential data to provide a more complete understanding of the results. Besides interviewing the ASBAs, their OT teams and supervisors were also interviewed, offering a broader perspective to the situation.

The sample size for the quantitative survey represented a significant portion of all graduates (39.7%). However, as it was not fully representative, the power of the analysis was weakened, and results may not represent all of the ASBA graduates. Possible reasons for no response include not seeing the email, change of email address since graduation, or lack of interest in participating. Additionally, the terms describing how often CSs were completed, ‘frequent’, ‘occasionally’, and ‘rare’, were subjective and not defined. This is why participants were additionally asked to estimate the number of CSs they performed monthly.

Many of the survey questions relied on memory and may result in recall bias or be inaccurate. Further, the absence of comprehensive health facility statistics and broader health outcomes data restricts the depth and breadth of the analysis. Given that functionality of an OT changes frequently with changing personnel in rural settings, and outcome data collection is often missing or inaccurate, health facility statistics were not able to be utilized.

For the qualitative data, only seven rural sites were selected for the in-depth interviews and focus group discussions. This selective sampling provided a broad range of perspectives from each geographical region, hospital level, and province while taking into consideration varying caseloads and population densities surrounding the health facility. This allowed for a broad range of perspectives, but the selective sampling cannot be nationally representative.

The wider literature supported the findings of this paper, but most of the research on surgical medical officers is based in sub-Saharan Africa, with vast cultural, geographical, and regional differences from Nepal, making their relevance questionable. More research is needed on the efficacy and outcomes of task-shifting in South Asia.

## Recommendations and policy implications

First and foremost, the ASBA training program should be continued to provide CS services in Nepal where obstetrician specialists are not readily available. The following specific features of the program and deployment of ASBA trainees should be considered:

**At a CEmONC Health Facility** ASBAs must be stationed at CEmONC health facilities with a functioning operating theater, not simply sent to health facilities expected to provide CEmONC. As of 2023, no policy dictates where ASBAs are to be stationed after training. This should be addressed to ensure that all ASBAs are placed where their skills can be successfully utilized.

**With a Senior Staff Member** The greatest predictive factor of an ASBA performing CSs was the presence of a family physician or another ASBA. If an ASBA was present at a health facility with multiple staff responsible for CSs, the burden of CSs on any individual was reduced and this increased the confidence of the ASBA graduates. In contrast to the presence of another ASBA or family physician, more than one OB/GYN was cited as the greatest hindering factor for the ASBAs who were not able to perform CSs at their health facility. Thus, hospitals with multiple OB/GYN should not be considered as deployment sites for ASBA graduates.

### At a primary, secondary A, or secondary B hospitals

OT teams, Me.Su.s, and ASBAs all recommended that ASBA graduates be placed in district hospitals with an adequate catchment area to ensure adequate exposure to cases. ASBA graduates were found to be significantly more likely to be performing CSs at Primary and Secondary A health facilities compared to Tertiary health facilities, when the presence of a functional OT room was taken into account (*p* = p = 0.005; *p* = 0.001). Multiple individuals stated that ASBAs were not useful in Tertiary health facilities, which may already have multiple gynecologists. Thus, ASBA graduates should not be placed at Tertiary health facilities, but rather in Primary and Secondary hospitals with a functional OT.

### ASBA curriculum recommendations

Emergency hysterectomies and safe abortion care were requested to be added to the ASBA training curriculum as well as greater exposure to complicated cases during training to increase the ASBA’s confidence. Further study of the case types encountered by ASBA graduates is needed to decide on how to best improve the ASBA training.

## Conclusion

The majority of ASBA graduates continue to provide CSs in remote and geographically difficult areas of Nepal, reducing HR shortages, expanding the provision of life-saving services, and improving the working conditions in rural hospitals within the LMIC setting. The ASBA program should be continued to further increase access to CSs and reduce maternal and infant mortality, especially in remote regions where specialist posts cannot be filled. However, ASBAs can only be successful if the appropriate health facility infrastructure is in place. This emphasizes the need for a holistic approach to solving complex global health problems and is a reminder that task-shifting alone is not the answer. The ASBA program should serve as a case study for successful surgical task shifting in LMICs.

## Supplementary Material

Supplemental Material

## Data Availability

The quantitative data is available for download here: https://datadryad.org/stash/share/KMF3JHiynbZMLep9x3XH3r-JPNeHm7627wHrGDOx5rA.
