# Supplementary figures and images for: Successful task shifting: a mixed-methods cross-sectional evaluation of an emergency obstetric care program to increase access to cesarean sections in rural Nepal
Source: Glob Health Action. 2024 Dec 3;17(1):2429888. doi: 10.1080/16549716.2024.2429888 (PMC11616743; doi:10.1080/16549716.2024.2429888)

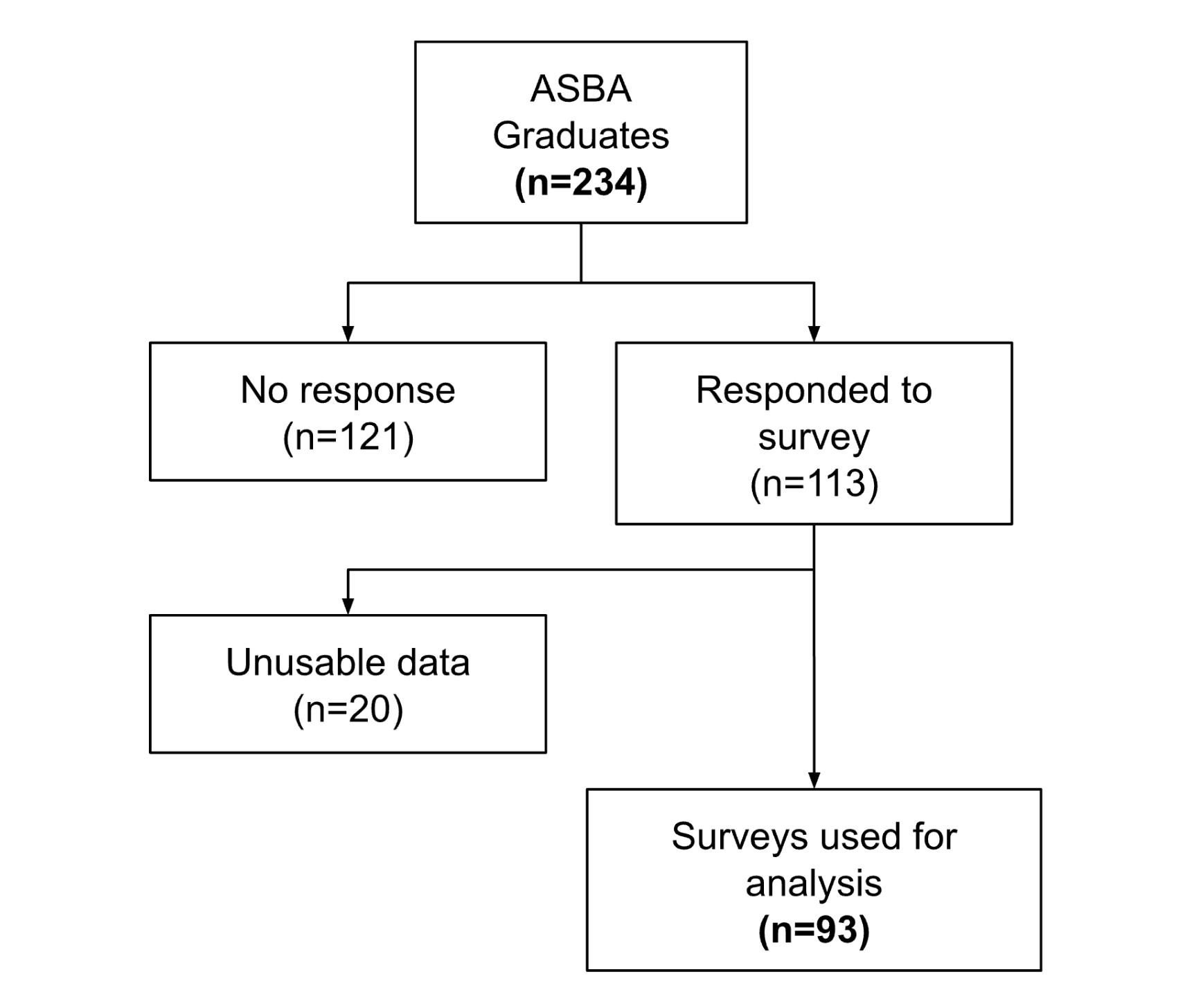

Supplement: Supplemental Material [file ZGHA_A_2429888_SM6035.zip › Supplementary Figures/Supplemental_Figure S1_ASBA_tiff.tiff]

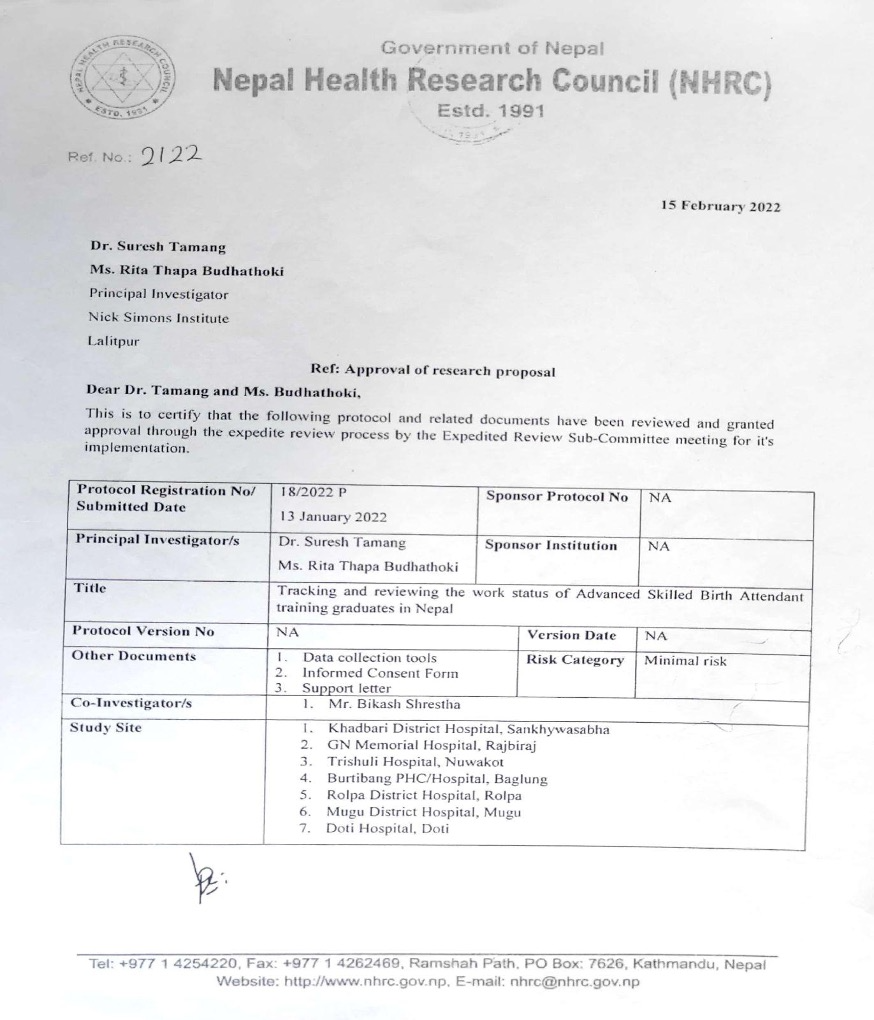

Supplement: Supplemental Material [file ZGHA_A_2429888_SM6035.zip › Supplementary Figures/Supplemental_Figure S2_ASBA_tiff.tiff]
